# Online Racial Discrimination and College Student Mental Health: Social Support as a Moderator

**DOI:** 10.1007/s40615-025-02405-w

**Published:** 2025-04-09

**Authors:** Ami Patel, Cixin Wang

**Affiliations:** Department of Counseling, Higher Education, and Special Education, University of Maryland, College Park, 3112E Benjamin Building, College Park, MD 20742 USA

**Keywords:** Online racial discrimination, Vicarious, Direct, College students, Social support, Mental health outcomes

## Abstract

Many college students experience racism and discrimination, which negatively impacts their mental health. As COVID-19 has brought about increased reliance on virtual spaces, increased online racial discrimination (ORD) has also become a more prevalent issue. ORD may be defined as online behaviors that demean or marginalize individuals or groups based on race. It may occur on social media, through text messaging, online videos/games, and/or online chat rooms. Research has shown that direct ORD predicted mental health difficulties, but the link between vicarious ORD and mental health was less consistent. Therefore, it is imperative to investigate how direct or vicarious ORD impacts student mental health. This study aims to examine the relationships between direct and vicarious ORD and college student mental health (depression, anxiety, flourishing, and health-related quality of life) and compare the relationships between White students and students of color. Data were collected from a sample of 617 undergraduate students (*M*_age_ = 19.5 years, *SD* = 1.9, 71.6% female) from two large, urban universities in the Mid-Atlantic region of the USA during COVID-19 from October to December 2021. Our findings revealed that both direct and vicarious ORD predicted depression and anxiety among students of color. However, this was not the case among White students. We also found that social support was a significant buffer on the relation between vicarious ORD and anxiety and flourishing. Social support was a buffer on the relation between direct ORD and health-related quality of life for students of color, but only at low levels of direct ORD. Our findings have implications for future intervention development.

College campuses, especially predominantly White campuses, are important settings where students encounter and learn about race and racism [22]. Racial discrimination remains prevalent on college campuses, even with the diverse representation of students from various socioeconomic and ethnic backgrounds in the USA. Racial discrimination is the unfair treatment of an individual based on the characteristics of race. As COVID-19 brought about increased reliance on virtual spaces, increased online racial discrimination (ORD) has also become a prevalent issue [7, 17, 31, 32]. We define ORD as “denigrating or excluding individuals or groups on the basis of race through the use of symbols, voice, video, images, text, and graphic representations” [59, p. 2]. It may occur on social media, through text messaging, online videos, online games, and/or online chat rooms. ORD is often anonymous, text-based, or interpersonal while in-person forms of racial discrimination can be verbal and/or physical [58]. Though growing, less research has focused on ORD or examining the impact of both direct and vicarious ORD experiences on mental health. Furthermore, very few studies have examined the impact of ORD on positive mental health outcomes [7, 57]. The dual factor of mental health [53] suggests mental health consists of two dimensions: mental illness symptoms as well as positive mental health/well-being. Not all young adults with clinical levels of psychopathology experience poor quality of life. On the other hand, some youth without any psychopathology/symptoms may struggle with well-being [53]. As a result, incorporating both positive and negative indicators of well-being into mental health assessments can provide a more comprehensive and nuanced understanding of one’s overall functioning [53].

Both direct and vicarious ORD have been associated with mental health difficulties like depression and anxiety, even when controlling for factors like ethnic/racial group and gender [59]. Direct racial discrimination involves a targeted assault on an individual’s ethnic identity [58]. The personalized nature of this racial discrimination can create immediate distress, a sense of powerlessness, and intensifying feelings of hopelessness and helplessness, which are likely to lead to negative mental health symptoms, such as depression and anxiety [31, 55, 57–60]. Vicarious ORD is observing racism happening online and/or on the news directed toward other individuals in their racial or ethnic group. Vicarious discrimination tends to impact individuals by exposing them to the discriminatory experiences faced by others. Observing discrimination against others can evoke strong empathetic responses, strengthen the sense of racial belonging, and cause collective stress [55]. While this vicarious experience can foster increased solidarity, it can also lead to feelings of vulnerability (e.g., anxiety) and reduced positive mental health (e.g., life satisfaction) [55, 58, 60]. Research has shown that direct ORD predicted mental health difficulties, but the link between vicarious ORD and mental health difficulties was less consistent [32, 36, 57, 58]. In addition, very few research examined the relation between ORD and positive mental health indicators and findings were inconsistent [7, 57].

This study aims to address these gaps by investigating the link between different types of ORD (direct and vicarious) along with depression and anxiety, as well as flourishing and health-related quality of life among a diverse sample of college students during the COVID-19 pandemic (between October and December 2021). It also explores the buffering effects of social support on these factors. Prior research has shown that social support can act as a protective factor, reducing the negative psychological impact of discrimination [31]. Understanding the impact of different types of ORD on health and well-being in college settings and identifying potential protective factors is crucial for addressing broader racial inequities among young adults [20].

## Discrimination Among White Americans

While researchers generally agreed that students of color frequently face racial discrimination, some studies have indicated White Americans also self-reported experiencing discrimination, with the rates of perceived discrimination on the rise [56]. For example, a Pew Research Center survey, including 12,055 US adults across categories such as gender, race, ethnicity, partisan affiliation, education, and other demographic factors, found that 14% of Americans in general believe White people experience a lot of discrimination [45]. Other studies found that White people perceived declining anti-Black discrimination and rising anti-White discrimination over time (e.g., from the 1950s to the present/2010s) [43]. These discrimination perceptions are moderated by White people’s political party affiliation: Republicans perceived more anti-White discrimination than Democrats and Independents [43]. Telhami and Rouse (2022) also found that White Americans believe they “have seen an increase in discrimination against other White people in the past five years.”

While White Americans may experience discrimination, such experiences are qualitatively different from racial discrimination and racism experienced by racial minorities. Researchers used longitudinal data across different domains and found Black-White educational disparities continue to persist in the twenty-first century due to a longstanding history of racial exclusion, which has restricted Black Americans’ access to homeownership, high-performing schools, higher education, and well-paying jobs [35]. White Americans may experience discrimination, but not racism, due to the power imbalance inherent in racism, with some researchers suggesting that discrimination against White people is often mild and less consequential because of their high status [33]. White Americans are the dominant racial group in the USA and hold great power in society [56] and may be claiming discrimination to maintain their dominant status and privileges when they perceive a decline in their social and economic status. This claim is related to their “sense of group threat in response to social and political changes” [40, p. 48]. “Racial resentment”—perceptions by White people that their status at the top of the American racial hierarchy is under threat by the rise of racial minorities [34]—also might contribute to the increase in Whites’ perceptions that their racial group might face discrimination [10, 18]. Further, cross-racial contact—increased contact with racial minorities—can trigger status anxiety among White people, predisposing White people to perceive racial discrimination in daily interactions [18, 34]. It may also explain why White people tend to report more discrimination in contexts where they are the numeric minority. One author describes the concept of “entitlement to racial comfort” [14]. For example, White people have not developed resilience to racial discomfort. Therefore, when they experience such discomfort, they may perceive the discomfort as abnormal and often attribute blame to the person or the situation that caused it, usually a person of color.

Few studies have directly examined this hypothesis and compared racial discrimination and its associated consequences among different racial groups. For example, data were collected from 64,041 undergraduate students participating in the Spring 2021 American College Health Association-National College Health Assessment [32]. Logistic regression showed that White students, as well as students of color, all reported COVID-19–related racial discrimination. COVID-19–related racial discrimination refers to both the direct and vicarious ways in which the pandemic has amplified and brought increased visibility to longstanding racial injustices, such as xenophobia, anti-Asian hate crimes, and verbal harassment. Additionally, as virtual communication surged during the pandemic, social media became a central platform for exposing and learning about instances of racial discrimination related to the pandemic [32]. COVID-19–related vicarious discrimination predicted psychological distress among White students as well as students of color, but COVID-19–related direct discrimination only predicted psychological distress among students of color [32]. Considering the different patterns among White students vs. students of color, it is important to further compare the experiences and consequences of different types of ORD among White students and students of color to better understand discrimination among different groups across different forms.

## Online Racial Discrimination and College Student Mental Health

Mental health problems are common among all college students. A national survey with data from 373 campuses across the USA showed that over 60% of college students met the criteria for at least one mental health disorder [29]. In another nationwide survey, nearly 75% of students disclosed experiencing moderate to severe psychological distress [1]. The pandemic exacerbated mental health challenges among college students [5]. Moreover, there has been increased discrimination against racial minorities during COVID-19. A significant percentage of Black (20%) and Asian (26%) adults feared others might threaten or physically attack them because of their race [48]. A report shared that racial minorities are more likely than their White counterparts to say they have experienced discrimination based on their race or ethnicity, though it did not specify the context in which this discrimination occurred (e.g., in-person, online) [48].

ORD represents a modern form of racism, as the perceived anonymity of the internet allows perpetrators to feel as though they are part of a group with little risk of being identified [31]. With internet usage among US adults increasing from 52% in 2000 to 93% in 2021 [44] and heightened exposure to news and social media [33], ORD is very prevalent among young people. For example, one study revealed that 29% of African American, 20% of White, and 42% of multiracial/other youth (*n* = 264, aged 14 to 18) reported experiencing direct ORD, while 71% of African American, 71% of White, and 67% of multiracial/other high school students reported experiencing vicarious ORD at least once. Another study showed that 58% of Latinx students reported at least one instance of both vicarious and individual OR/ED in the past year [46]. Direct and vicarious experiences of racism have been associated with mental health. For example, research among Black college students (*n* = 278) found that ORD was associated with depression and generalized anxiety (but not social anxiety) [17]. Similar results were noted between both direct and vicarious ORD and anxiety and depressive symptoms among Latinx undergraduates (*n* = 522) [46]. A longitudinal study among African American and Latinx adolescents in grades 6 through 12 over 3 years showed that ORD experiences increased overtime for most participants, and both direct and vicarious ORD were related to depression, anxiety, and self-esteem [58].

However, other studies showed only online direct racial discrimination impacted psychological outcomes. Higher levels of online direct discrimination (but not vicarious discrimination) were significantly associated with increased depression and anxiety among a diverse sample of high school students (*n* = 264, aged 14 to 18) [57]. Similar to this, online direct racial discrimination (not vicarious discrimination) predicted negative perception of campus racial climate among African American and White college students (*n* = 217) [60]. Regarding positive mental well-being, some researchers found that online racial discrimination was negatively related to lower overall well-being [7], while another study showed that ORD was not related to positive mental health outcomes (i.e., satisfaction with life) [57].

It is crucial to understand how discrimination manifests in online settings [46] given ORD is becoming more prevalent and is expected to have a particularly pronounced psychological impact [31]. Overall, the research highlights the detrimental effects of direct ORD on the mental health of college students, particularly students of color. However, the impact of vicarious ORD is less clear in the literature. Furthermore, many studies have centered on negative mental health outcomes such as depression and anxiety, neglecting the impact of ORD on positive aspects of mental well-being such as flourishing or quality of life. Understanding these dynamics is key for developing effective interventions and support systems within college campuses.

## Social Support and College Student Mental Health

Social support, which refers to the presence or availability of others (e.g., family, friends) who express concern, love, and care and provide coping assistance, is considered an important resource [40] for individuals who experience and assess a discriminatory experience as a threat or challenge. Social support is measured by individuals’ perception of the adequacy of support they receive from these sources, such as comforting messages, resources, and advice that can help individuals cope. By seeking support from family, friends, and loved ones, individuals realize they are not alone and may gain access to more effective coping mechanisms, including emotion-focused and problem-focused strategies, which can help reduce psychological distress.

Research highlights the importance of social support, particularly from family and peers, for college students’ mental health. Peer interactions, shaped by contexts like classes and extracurricular activities, influence students’ interpretations of their environment (e.g., discrimination) and coping strategies (e.g., social support) [12]. One study found that only support from friends (not family or romantic partners) alleviated stress and loneliness in 636 college students self-identifying as White (53%), Hispanic (21.7%), Black or African American (13.4%), Asian American (3.6%), multiracial (6.4%), and as other (1.9%), although support from friends or romantic partners was linked to lower levels of loneliness, regardless of stress levels [27]. Other research shows that lack of support is tied to greater anxiety, loneliness, and depression [23, 41].

### Social Support as a Moderator

Social support plays a crucial role in promoting a sense of security, connectedness, and belonging when individuals experience racial discrimination. It can provide a shared understanding of the experience and help individuals cope with the stressor [4, 28]. Having a supportive social network can lead individuals to participate in social activities related to their support system, distracting them from stressors and inducing positive feelings to buffer the negative consequences of the discrimination experienced [4]. Research has demonstrated that social support can buffer the detrimental impact of racial discrimination on vascular reactivity among Black Americans [9] and psychological well-being for Asian Americans [65]. However, there are mixed findings. A meta-analytic review by Schmitt et al. [49] found that the moderating effect of social support was not significant in many studies. The relationship between social support and mental health outcomes appears to be influenced by the different types of discrimination studied [31, 42]. Some studies have shown that social support consistently acts as a buffer against psychological symptoms in college students who have experienced direct, overt racial discrimination [31]. On the other hand, some research showed that social support did not function as a stress buffer for vicarious racial discrimination [31, 49]. The effectiveness of social support as a buffer may also depend on the level of racism-related stress. When racism-related stress is low, social support may be more effective in mitigating the negative effects of discrimination, particularly for Asian Americans and African Americans. However, when racism-related stress is high and discrimination is frequent, the influence of social support may be limited [9], possibly because racism can become overwhelming when it occurs very frequently [65]. Further research is warranted to better understand the nuances of social support in coping with different types of ORD (direct vs. vicarious) for different racial and ethnic groups.

## Current Study

The proposed study aims to examine the relationship between online direct and vicarious racial discrimination and college student mental health among White students vs. students of color. It also aims to investigate the potential moderating role of social support in this relationship. Based on the existing literature, the study hypothesizes that ORD will predict poorer mental health outcomes among college students such that those who experience ORD (either direct or vicarious) will have higher rates of depression and anxiety and lower rates of flourishing (e.g., self-perceived success in areas such as relationships, self-esteem, purpose, and optimism) and health-related quality of life. We will also compare the relationship between ORD and mental health separately for White students and students of color.

Furthermore, the study proposes that social support will buffer the negative effects of ORD on college student mental health. This hypothesis is based on the idea that social support plays a crucial role in navigating discriminatory experiences and coping with them, particularly considering the college environment and the influence of peers, family, and significant others [2, 4, 12].

## Methods

### Procedure

The study received approval from the Institutional Review Board. Data were collected from undergraduate students at two large, urban universities in the Mid-Atlantic region of the USA. Both of these universities are racially diverse, with White students making up about 40% of the student body [39]. An anonymous survey link was distributed to Sociology and Psychology 101 courses, as well as university listservs created by the registrar’s office. Participants had the option to receive extra credits for Sociology or Psychology 101 courses or enter a drawing for a $20 Amazon gift card upon survey completion. Data collection took place from October to December 2021.

### Participants

The sample for this study consisted of 617 undergraduate college students (*M*_age_ = 19.5 years, *SD* = 1.9, 71.6% identified as female and 25.6% as male). In terms of grade level, 41.8% were freshmen (*n* = 258), 25.1% were sophomores (*n* = 155), 16.2% were juniors (*n* = 100), and 15.9% were seniors (*n* = 98). Regarding race/ethnicity, 55.1% of participants identified as White (*n* = 340), 24% as Asian (*n* = 148), 10% as Black or African American (*n* = 62), 4.2% as Latino/Hispanic/Mexican (*n* = 26), 1.9% as Middle Eastern/Arab (*n* = 12), and 0.3% as American Indian or Alaska Native (*n* = 2).

### Measures

#### Direct and Vicarious Online Racial Discrimination

The Individual Online Racial Discrimination subscale from the Online Victimization Scale (OVS) [61] was used to measure direct ORD targeting the participants. The subscale consists of three items and the average total score was computed (e.g., “People have said mean or rude things about me because of my race or ethnic group online”). The vicarious ORD subscale measures ORD targeting the participants’ race/ethnic group in general, also consisting of three items, and the average total score was computed (e.g., “People have said things that were untrue about people in my race or ethnic group online”). For both subscales, participants rated how often they experienced each incident on a 5-point scale—1 (*never*), 2 (*about once a month or less*), 3 (*a few times a month*), 4 (*once or twice a week*), and 5 (*almost every day*). Prior studies showed this measure had high internal consistency ranging from 0.66 to 0.92 [54, 57, 58] and good validity [56]. Internal consistency for the subscale of direct ORD in the current study was 0.72 and 0.92 for the subscale of vicarious ORD.

#### Depressive Symptoms

Depressive symptoms were measured using the 9-item Patient Health Questionnaire (PHQ-9) [24]. Participants rated items (e.g., “little interest or pleasure in doing things”) on a 4-point scale ranging from 1 (*not at all*) *to* 4 (*nearly every day*). Total scores on the PHQ-9 were used, and different cutoff ranges were used to categorize the severity of depressive symptoms: 10–14 (moderate), 15–19 (moderately severe), and 20–27 (severe) [62]. Internal consistency for the PHQ-9 was shown to be excellent (*α* = 0.89) in primary care settings along with a variety of other populations (*α* = 0.82–0.91) [8, 54]. Internal consistency in the current study was 0.90.

#### Anxiety Symptoms

Generalized anxiety symptoms were assessed using the 7-item Generalized Anxiety Disorder Screener (GAD-7) [52]. The average summary score of this measure was computed. Participants were asked to indicate how often they had been bothered by problems such as “worrying too much about different things” or “trouble relaxing” over the past 2 weeks, using a 4-point scale ranging from 1 (*not at all*) to 4 (*nearly every day*). Total scores on the GAD-7 can range from 0 to 21, with higher scores indicating greater general anxiety symptoms. A clinical threshold of 10 is recommended for moderate anxiety [21]. The GAD-7 has demonstrated high internal consistency (*α* = 0.82–0.89) [21, 30], clinical utility, and construct validity with the general population [26], as well as excellent validity [30]. Internal consistency in the current study was 0.92.

#### Flourishing

The Flourishing Scale is an 8-item measure that assesses the respondent’s self-perceived success in important areas such as relationships, self-esteem, purpose, and optimism [15]. Participants rate their agreement with statements such as “I lead a purposeful and meaningful life” and “I am optimistic about my future” on a 7-point Likert scale from 1 (*very strongly disagree*) to 7 (*very strongly agree*). Higher scores indicate greater psychological resources and strengths. This scale has been validated in other populations and has produced high reliability (*α* from 0.82 to 0.90) [11, 16, 63]. Internal consistency in the current study was 0.93.

#### Health-Related Quality of Life

The Health-Related Quality of Life–Short Form (SF-8) is a widely used measure for assessing health-related quality of life (HRQOL), including general health, physical functioning, role physical and emotional, bodily pain, vitality, social functioning, and mental health. A sample item includes “During the past 4 weeks, how much energy did you have?” Items were reverse-coded so that a higher endorsement of items indicates better health-related quality of life. The SF-8 has demonstrated good reliability in previous studies ranging from 0.73 to 0.92 and has been well-validated among a variety of populations [3, 25]. Internal consistency in the current study was 0.85.

#### Social Support

The Multidimensional Scale of Perceived Social Support (MSPSS) [66] items were combined into four items (from twelve items) for this study (e.g., “My friend(s)/family/other(s) really try to help me,” “I have a friend(s)/family/other(s) I can share sorrow and joys with”). The average score of the four items was calculated to create a composite score [37]. Participants rated their perceived support from family, friends, or others on a 7-point Likert scale ranging from 1 (*very strongly disagree*) to 7 (*very strongly agree*). Previous studies have shown good internal consistency for both subscale and total scores of the MSPSS, typically ranging from 0.81 to 0.94. The scale has also demonstrated good validity [50]. Internal consistency in the current study was 0.93.

### Data Analyses

Using SPSS 29.0, we conducted an analysis of variance (ANOVA) to investigate ethnic group differences for online direct and/or vicarious racial discrimination. We then performed separate linear regression analyses using SPSS 29.0 to examine the relationships between online direct and vicarious racial discrimination on college student mental health, specifically for depression, anxiety, health-related quality of life, and flourishing. Gender (male = 1) and grade levels were used as control variables in the regression analysis. Additionally, linear regression analyses were conducted to explore differences in racial discrimination and college student mental health among White students vs. students of color (White = 1, students of color = 0). To address the second research question, moderation analyses were conducted to examine whether social support moderated the relationship between ORD and college student mental health. Before analysis, predictors and moderators were mean-centered. In cases of significant interaction, Microsoft Excel was used to graph the simple slope analysis to further explore the nature of the interaction. Descriptive statistics were also computed for all variables in the study.

## Results

### Preliminary Data and Correlations

Of the 617 undergraduate college students recruited, results suggested that ORD among college students was prevalent. Particularly for White students in our sample (*n* = 335), 55% reported experiencing online direct racial discrimination (“about once a month” or more), and 18.5% reported experiencing online vicarious racial discrimination (“about once a month” or more). On the other hand, among the participants who identified as students of color (*n* = 282), 50% of them reported experiencing online direct racial discrimination (“about once a month” or more), with 86% having reported experiencing online vicarious racial discrimination (“about once a month” or more). However, on average, students of color reported experiencing higher levels of both online direct racial discrimination (*t* = 6.41, *p* < 0.001) and online vicarious racial discrimination (*t* = 11.10, *p* < 0.001) compared to White students, even though more than half of White students also reported experiencing online direct racial discrimination.

Moreover, our ANOVA findings indicated that Asian and Black college students reported experiencing more online direct racial discrimination and vicarious racial discrimination than White college students (*p* < 0.001). Latinx/Hispanic students also reported experiencing online vicarious racial discrimination more than their White counterparts (*p* < 0.001). Black students reported experiencing more online vicarious racial discrimination than Asian college students (*p* < 0.001) (see Table 1). The majority of participants in the study presented with anxiety and/or depressive symptoms, suggesting a high prevalence of mental health symptoms during COVID-19. Specifically, 64.2% of students reported significantly elevated depressive symptoms (above the cutoff score of PHQ-9) while 61.1% of students reported significantly elevated anxiety (above the cutoff score of GAD-7). All variables of interest were significantly correlated with one another (see Table 2).

**Table 1:** Mean differences by racial group

	American Indian or Alaska Native	Asian	Black or African American	White	Middle Eastern/Arab	Latino/Hispanic/Mexican
Online direct discrimination	1.17	1.35***	1.48***	1.14	1.31	1.32
Online vicarious discrimination	2.33	2.61***	3.31***	1.75	2.33	2.73***
Depression	1.33	1.94	2.17*	1.86	2.11	1.91
Anxiety	1.57	2.14	2.26	2.17	2.38	2.07
Flourishing	5.94	5.34***	5.33	5.69	5.26	5.62
Health-related quality of life	2.06	2.42	2.45	2.46	2.58	2.44

^*^Being significantly different compared with White students. *p < .05, **p < .01, ***p < .001

**Table 2:** Descriptive statistics for all students

	1	2	3	4	5	6	7
1. Online vicarious RD	-						
2. Online direct RD	.50**	-					
3. Depression	.18**	.18**	-				
4. HRQOL	.08*	.10*	.66**	-			
5. Flourishing	− .17**	− .13**	− .59**	.45**	-		
6. Anxiety	.09*	.11**	.76**	.63**	− .38**	-	
7. Social support	− .13**	− .13**	− .28**	.18**	.52**	− .12**	-
Mean	2.22	1.25	1.93	2.45	5.54	2.18	5.88
SD	1.24	0.47	0.72	0.70	1.06	0.82	1.17
*n*	617	617	617	617	617	617	617

*RD* racial discrimination, *HRQOL* health-related quality of life

^*^p < .05, **p < .01, ***p < .001

### Factors Predicting Mental Health Outcomes

Regression analysis showed that online direct racial discrimination (*β* = 0.11, *t*(611) = 2.514, *p* = 0.01) and online vicarious racial discrimination (*β* = 0.09, *t*(611) = 2.242,* p* = 0.03) significantly predicted more depression (Table 3). Online direct racial discrimination (*β* = 0.09, *t*(611) = 2.18, *p* = 0.03) and social support (*β* = − 0.13, *t*(611) = − 3.37, *p* < 0.001) were related to anxiety symptoms in college students. Significant main effects were present for online vicarious racial discrimination (*β* = − 0.09, *t*(611) = − 2.48, *p* = 0.01) and social support (*β* = 0.51, *t*(611) = 14.55, *p* < 0.001) for flourishing (Table 4). Lastly, social support (*β* = 0.20, *t*(611) = 5.09, *p* < 0.001) predicted health-related quality of life.

**Table 3:** Social support moderation between online racial discrimination and depression and anxiety

	Depression				Anxiety			
	Main effect model		Interaction model		Main effect model		Interaction model	
	*b*(SE)	*β*	*b*(SE)	*β*	*b*(SE)	*β*	*b*(SE)	*β*
Constant	1.91(0.06)***		1.91(0.06)***		2.26(0.07)***		2.26(0.07)***	
Online vicarious	0.07(0.03)*	0.09*	0.06(0.03)*	0.09*	0.03(0.04)	0.03	0.008(0.04)	0.01
Online direct	0.08(0.03)**	0.11**	0.09(0.03)**	0.12**	0.08(0.04)*	0.09*	0.10(0.04)**	0.12**
Social support	− 0.2(0.03)***	− 0.28***	− 0.2(0.03)***	− 0.28***	− 0.11(0.03)***	− 0.13***	− 0.11(0.03)***	− 0.13***
Male	− 0.32(0.06)***	− 0.19***	− 0.32(0.06)***	− 0.19***	− 0.43(0.07)***	− 0.23***	− 0.43(0.07)***	− 0.23***
Grade level	0.05(0.02)*	0.08*	0.046(0.02)*	0.07*	0.01(0.03)	0.02	0.01(0.03)	0.01
Online vicarious* social support			− 0.05(0.03)	− 0.07			− 0.11(0.04)**	− 0.14**
Online direct* social support			0.03(0.023)	0.05			0.06(0.03)	0.09
*F*(df)	21.65(5,611)***		15.92(7,609)***		10.32(5,611)***		8.86(7,609)***	
*R*^2^	0.15		0.16		0.08		0.09	
Δ*R*^2^			0.004				0.02	

^*^p < .05, **p < .01, ***p < .001

**Table 4:** Social support moderation between online racial discrimination and flourishing and quality of life

	Flourishing				HRQOL			
	Main effect model		Interaction model		Main effect model		Interaction model	
	*b*(SE)	*β*	*b*(SE)	*β*	*b*(SE)	*β*	*b*(SE)	*β*
Constant	5.51(0.08)***		5.52(0.08)***		4.45(0.06)***		4.45(0.06)***	
Online vicarious	− 0.10(0.04)**	− 0.09**	− 0.10(0.04)**	− 0.09**	− 0.02(0.03)	− 0.03	− 0.02(0.03)	− 0.02
Online direct	− 0.02(0.04)	− 0.02	− 0.01(0.04)	− 0.01	− 0.05(0.03)	− 0.07	− 0.06(0.03)*	− 0.09*
Social support	0.54(0.04)***	0.51***	0.52(0.04)***	0.49***	0.14(0.03)***	0.20***	0.15(0.03)***	0.21***
Male	0.02(0.08)	0.01	0.00(0.08)	0.00	0.40(0.06)***	0.25***	0.41(0.06)***	0.26***
Grade level	0.01(0.03)	0.01	0.02(0.03)	0.02	− 0.00(0.02)	− 0.01	− 0.00(0.02)	− 0.01
Online vicarious* social support			0.08(0.04)*	0.08*			0.02(0.03)	0.03
Online direct* social support			0.05(0.04)	0.06			− 0.05(0.023)	− 0.08
*F*(df)	48.16(5,611)***		36.56(7,609)***			13.91(5,611)***		10.45(7,609)***
*R*^2^	0.28		0.29			0.10		0.11
Δ*R*^2^			0.01					0.01

*HRQOL* health-related quality of life

^*^p < .05, **p < .01, ***p < .001

In addition, two separate sets of regression models were run to compare the results of White students and students of color in the study. The regression model in Table 5, particularly for students of color, showed online direct and vicarious racial discrimination predicted depression and anxiety but did not predict flourishing or health-related quality of life. For White students, online direct and vicarious racial discrimination did not significantly predict any of the mental health outcomes.

**Table 5:** White students vs. students of color reported experiences of racial discrimination predicting mental health outcomes

	Depression		Anxiety		Flourishing		Health-related quality of life	
	*b*(SE)	*β*	*b*(SE)	*β*	*b*(SE)	*β*	*b*(SE)	*Β*
White								
Constant	1.726(0.142)***		2.232(0.165)***		6.016(0.207)***		2.258(0.143)***	
Grade level	0.03(0.03)	0.051	0.008(0.038)	0.011	0.018(0.047)	0.021	0.004(0.033)	0.007
Online vicarious	0.012(0.043)	0.017	− 0.058(0.05)	− 0.074	− 0.076(0.063)	− 0.078	0.051(0.043)	0.073
Online direct	0.093(0.12)	0.049	0.109(0.139)	0.049	− 0.201(0.175)	− 0.074	0.193(0.121)	0.098
Male	− 0.248(0.092)**	− 0.15**	− 0.447(0.107)***	− 0.228***	− 0.024(0.134)	− 0.01	− 0.506(0.093)***	− 0.292***
Students of color								
Constant	1.298(0.151)***		1.571(0.172)***		5.829(0.237)***		2.231(0.143)***	
Grade level	0.074(0.037)*	0.11*	0.01(0.042)	0.013	− 0.024(0.059)	− 0.024	0(0.035)	0
Online vicarious	0.106(0.037)**	0.175**	0.125(.042)**	0.185**	− 0.106(0.059)	− 0.119	0.045(0.035)	0.083
Online direct	0.246(0.086)**	0.175**	0.235(0.098)*	0.149*	− 0.059(0.135)	− 0.028	0.105(0.081)	0.082
Male	− 0.257(0.093)**	− 0.157**	− 0.274(0.105)**	− 0.15**	− 0.151(0.145)	− 0.063	− 0.238(0.088)**	− 0.161**

^*^p < .05, **p < .01, ***p < .001

### Social Support as a Buffer for Some Mental Health Outcomes

**Fig. 1:**
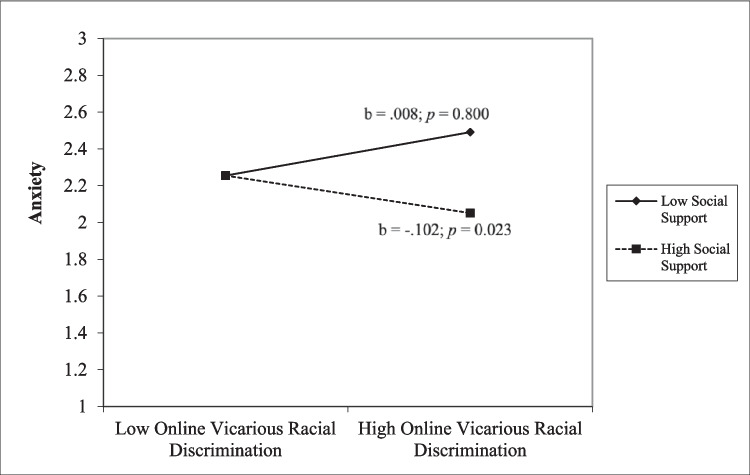
Simple slope analysis for interaction of online vicarious racial discrimination and anxiety

**Fig. 2:**
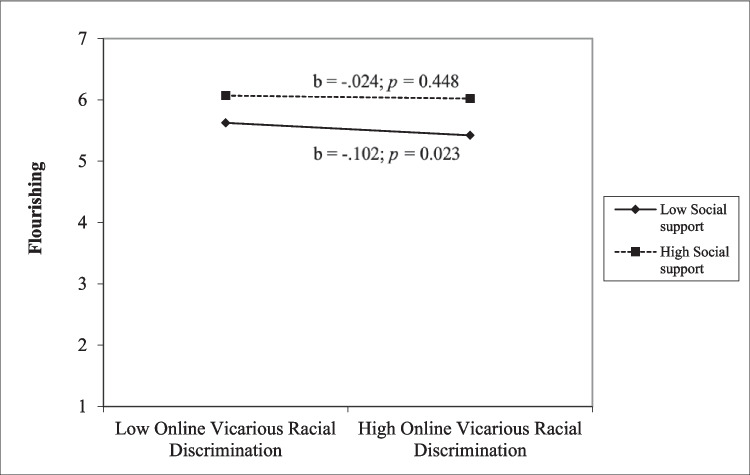
Simple slope analysis for interaction of online vicarious racial discrimination and flourishing

After adding the interaction terms to the main effect models, we found that social support was a buffer for vicarious racial discrimination on anxiety for the whole sample (*β* = − 0.14, *t*(609) = − 3.09, *p* = 0.002) (Table 3). We then conducted a simple slope analysis. At high levels of online vicarious racial discrimination, students with higher levels of social support reported lower levels of anxiety than students with lower levels of social support (Fig. 1). Further, social support buffered the effect of online vicarious racial discrimination on flourishing for the whole sample (*β* = 0.08, *t*(609) = 1.94, *p* < 0.05). Similarly, a simple slope analysis was conducted. Online vicarious racial discrimination predicted lower levels of flourishing for students who reported lower social support (*b* = − 0.102, *p* = 0.02), but not for students who reported higher social support (*b* = − 0.024, *p* = 0.44; Fig. 2). The same pattern (social support as a buffer for vicarious ORD and anxiety and flourishing) was also identified for White students.

**Fig. 3:**
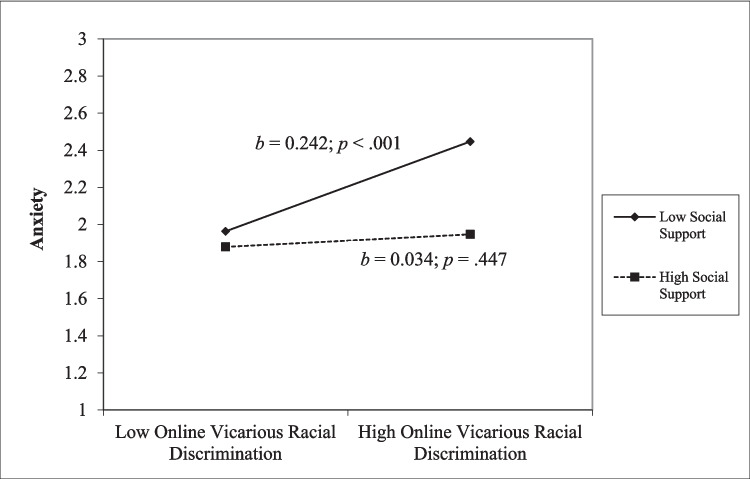
Simple slope analysis for interaction of online vicarious racial discrimination and anxiety for students of color

**Fig. 4:**
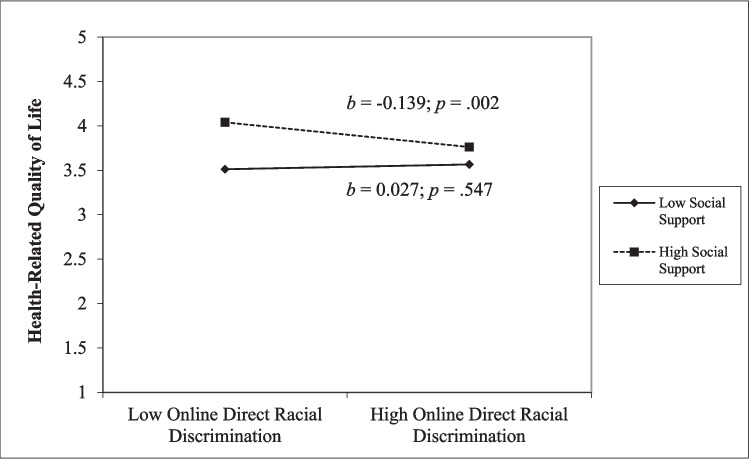
Simple slope analysis for interaction of online direct racial discrimination and health-related quality of life for students of color

However, the moderation results were somewhat different for students of color (Table 5). We found that social support was a buffer for online vicarious racial discrimination on anxiety for students of color (*β* = − 0.104, *t*(276) = − 2.18, *p* = 0.030). The simple slope analysis demonstrated that online vicarious racial discrimination predicted high levels of anxiety for students who reported lower levels of social support (*b* = 0.242, *p* < 0.001), but not for students with higher levels of social support (*b* = 0.034, *p* = 0.447; Fig. 3). Social support also moderated the effect of online direct racial discrimination on health-related quality of life, *β* = 0.14, *t*(281) = 2.01, *p* = 0.046. The simple slope analysis revealed that social support was a buffer for students’ quality of life at lower levels of direct ORD, but not at higher levels of direct discrimination (Fig. 4).

## Discussion

Research has shown that direct ORD predicted mental health difficulties, but the link between vicarious ORD and mental health difficulties was less consistent. In addition, very few research examined the relation between ORD and positive mental health indicators and findings were inconsistent. This study contributes to the literature by examining the relation between both vicarious and direct forms of ORD and both positive and negative indicators of mental health, including depression, anxiety, flourishing, and health-related quality of life, as well as the moderating role of social support. Additionally, to compare the differential impact of discrimination on mental health among different ethnic groups, we examined the relationship between ORD and mental health outcomes separately for White students and students of color. Consistent with prior research [31], we found many students of color (50%—direct, 86%—vicarious) experience high levels of discrimination, which warrants attention and intervention. On the other hand, a large percentage (55%—direct, 18.5%—vicarious) of White students in this study reported experiencing racial discrimination (although less frequent compared with students of color). More importantly, our findings added to the literature by illuminating the distinct mental health impact and the understanding of racial discrimination is different for White students (a dominant, unoppressed group) versus students of color (oppressed groups). Specifically, we found that racial discrimination did not predict any mental health difficulties among White students, although it predicted mental health difficulties among students of color. Finally, we found social support to be a buffer for most mental health outcomes that were investigated.

### Online Racial Discrimination Related to Mental Health Outcomes

It is important for research to examine the relationship between vicarious ORD and mental health outcomes given the growing reliance on online spaces, social media, and the increase in mental health difficulties [6, 47]. Our findings help differentiate the relations between online direct vs. vicarious racial discrimination and different mental health indicators. We found that vicarious ORD predicted both depression and flourishing beyond direct ORD. On the other hand, direct ORD predicted both depression and anxiety. This different pattern could be due to the direct and individual nature of racial discrimination targeted at participants versus the indirect psychological impact of vicarious racial discrimination. During COVID-19, social interactions were limited, and students highly relied on online interaction with their peers and the outside world. In our study, online vicarious racial discrimination was more prevalent than direct discrimination. The pervasive nature of students’ vicarious racial discrimination, the increased exposure to racial hate during the COVID-19 pandemic between October and December 2021, as well as greater reliance on online spaces, may have gradually undermined students’ well-being and positive resources [36]. Direct ORD is more related to negative outcomes/mental illness symptoms (e.g., depression and anxiety) possibly because it is more direct and hence harmful. Our findings of the differential effect of online direct and vicarious racial discrimination have extended our understanding of different ORD and may have implications for future intervention development targeting different types of ORD.

### Online Racial Discrimination Predicts Mental Health Outcomes Only for Students of Color

Online direct and vicarious racial discrimination was related to anxiety and depressive symptoms among students of color, but not among White students despite a large total percentage of White students reporting experiencing ORD. Our finding is partially consistent with another recent study [32] showing that COVID-19–related direct racial discrimination only predicted psychological distress among students of color, but COVID-19–related vicarious discrimination predicted psychological distress among both White students and students of color. Our study differs from Macaranas et al. (2023) in that they measured discrimination “as a result of the COVID-19 pandemic” and assessed general discrimination, whereas we specifically examined ORD. These methodological differences may help explain the contrasting findings between our study and theirs regarding vicarious discrimination.

Our finding of distinct mental health impacts of racial discrimination for White students versus students of color suggests that, while White students did experience discrimination, it appeared to be mild, less consequential, and did not significantly impact their mental health. This may be attributed to their higher social status and privilege. As described earlier, racial resentment can refer to White individuals’ perceptions that their position at the top of the American racial hierarchy is threatened by the growing presence of racial minorities [18, 34] which may contribute to the increasing belief among White individuals that their racial group is facing discrimination [10, 18]. Moreover, with the growing presence of racial minorities, frequent interactions with racial minorities may trigger status anxiety that can influence their likelihood of perceiving racial discrimination [18, 34]. This dynamic may also explain why White individuals report higher levels of discrimination in situations where they are in the minority. Our finding has implications for understanding White fragility and White privilege and for designing different programs to support students who experience racial discrimination in predominantly White institutions.

### Social Support as a Moderator for Online Vicarious and Direct Racial Discrimination

Extending prior research, we found that the effect of social support varied based on different types of ORD (direct vs. vicarious), student ethnicity (Whites vs. students of color), and on the specific mental health outcome studied. Social support buffered the effect of vicarious ORD on anxiety and flourishing for the whole sample and for White students. Social support only buffered the relationship between vicarious ORD and anxiety (not flourishing) for students of color. This finding is consistent with prior research [4, 9, 41]. When students experience vicarious ORD, having access to social support from their networks (e.g., family and friends) can better equip them to cope with stress. Social support may allow individuals to regulate negative thoughts and feelings associated with vicarious ORD by offering emotional support, practical assistance, and effective coping strategies [31]. Social support can also increase a sense of belonging and help individuals realize that they are not alone despite vicarious ORD. College students are spending more time online and are more likely to be exposed to vicarious ORD, so it is important to promote social support on college campus [19].

Regarding direct ORD, the findings are more complex. Our results suggested that social support was a buffer for quality of life only at low levels of direct ORD for students of color, but not at high levels of direct ORD, nor for the whole sample. It is possible that direct ORD became too overwhelming at high levels of racism, and social support was not sufficient to buffer its negative impact. This is consistent with prior research showing that social support may be more effective in mitigating the negative effects of discrimination when racism-related stress is lower [9, 65]. In addition, social support did not serve as a buffer for direct ORD for anxiety, depression, and flourishing. Other protective factors like cultural socialization and racial/ethnic identity may be a stronger buffer for college students, especially for direct ORD and for students of color. Cultural socialization, or the exposure to traditions, events, and holidays, can foster cultural pride in youth and has been shown to improve the psychosocial well-being of youth of color [13]. Cultural socialization can help students of color develop pride and resilience amid ethnic/racial discrimination [64]. Racial/ethnic identity has also been found to be a buffer for racial discrimination. A positive ethnic identity—being connected to and having positive attitudes toward one’s racial group—can provide students of color a range of strategies for coping with discriminatory experiences [51]. For example, individuals with positive ethnic identity can use positive views of their ethnic group and their sense of belonging to their group to discount ORD, which protects them from the adverse effects of discrimination [38].

### Limitations and Future Directions

There are several limitations that should be considered when interpreting the findings of this study. Firstly, the study design was cross-sectional, which prevents the establishment of causal relationships. Secondly, all data relied on self-report measures, which may be susceptible to biases, social desirability, and the mono-method bias. Future studies may consider longitudinal or experimental designs to examine the long-term impact of racial discrimination on mental health. Using a mixed-method approach, such as combining qualitative interviews with surveys, can allow for richer, more comprehensive data. For example, one can conduct focus group interviews with students from similar ethnic backgrounds to further understand the psychological effects of racial discrimination. Additionally, in combination with other measures, peer reports may provide valuable data, particularly when assessed at a campus-level or group level, capturing broader patterns of discriminatory behavior within a specific community.

Moreover, the study sample consisted solely of undergraduate students from two large universities in the Mid-Atlantic region, limiting the generalizability of the findings to other student populations from other regions. Furthermore, our study grouped students of color together due to the small sample size. Students of color represent very diverse subgroups, and each subgroup may have different experiences of ORD. It is crucial to include a more diverse sample of different subgroups of students to gain a comprehensive understanding of race-related online victimization and its impact on mental health. Future research may consider doing this by establishing collaborations with a broader range of universities, including community colleges, technical schools, and/or historically Black colleges and universities, to increase the diversity of participants and account for regional differences in experiences. Finally, it would have also been valuable to collect information on the specific types of online racial discrimination from different online sources to examine their different impacts on student mental health.

## Data Availability

The data are available from the corresponding author upon reasonable request.
